# Ghrelin Cells in the Gastrointestinal Tract

**DOI:** 10.1155/2010/945056

**Published:** 2010-03-14

**Authors:** Ichiro Sakata, Takafumi Sakai

**Affiliations:** ^1^Divisions of Hypothalamic Research and Endocrinology & Metabolism, Department of Internal Medicine, The University of Texas Southwestern Medical Center, 5323 Harry Hines Blvd, Dallas, TX 75390-9077, USA; ^2^Area of Regulatory Biology, Division of Life Science, Graduate School of Science and Engineering, Saitama University, Saitama 338-8570, Japan

## Abstract

Ghrelin is 28-amino-acid peptide that was discovered from the rat and human stomach in 1999. Since the discovery of ghrelin, various functions of ghrelin, including growth hormone release, feeding behavior, glucose metabolism, memory, and also antidepressant effects, have been studied. It has also been reported that ghrelin in the gastrointestinal tract has an important physiological effect on gastric acid secretion and gastrointestinal motility. Ghrelin has a unique structure that is modified by *O*-acylation with n-octanoic acid at third serine residues, and this modification enzyme has recently been identified and named ghrelin *O*-acyl transferase (GOAT). Ghrelin is considered to be a gut-brain peptide and is abundantly produced from endocrine cells in the gastrointestinal mucosa. In the gastrointestinal tract, ghrelin cells are most abundant in the stomach and are localized in gastric mucosal layers. Ghrelin cells are also widely distributed throughout the gastrointestinal tract. In addition, abundance of ghrelin cells in the gastric mucosa is evolutionally conserved from mammals to lower vertebrates, indicating that gastric ghrelin plays important roles for fundamental physiological functions. Ghrelin cells in the gastrointestinal tract are a major source of circulating plasma ghrelin, and thus understanding the physiology of these cells would reveal the biological significance of ghrelin.

## 1. Introduction

Several distinct types of endocrine cells, including histamine-producing ECL cells, somatostatin-producing D cells, gastrin-producing G cells, and serotonin-producing EC cells, have been shown to be present in the mammalian stomach. Before the discovery of ghrelin, gastric ghrelin cells were classified as unknown endocrine cells by their round, compact, electron-dense secretory granules (P/D(1)-type in humans, A-like-type in rodents, and X-type in dogs) that distinguish them electron-microscopically from other previously characterized gastric endocrine cell types [1, 2]. Ghrelin was identified as an endogenous ligand for growth hormone secretagogue receptor (GHS-R) from the stomach in 1999 [3]. Ghrelin structurally resembles motilin and they share about 21% amino acid identity and their receptor also has structural similarity with 44% similarity in amino acids, indicating that they comprise a motilin-ghrelin family [4, 5]. Interestingly, ghrelin was found to be present as two molecular forms: acyl-ghrelin modified with medium chain fatty acids and des-acylated-ghrelin lacking side chain modification [3, 6]. Although acyl-ghrelin is only known to bind to GHS-R [7], accumulating results have shown that des-acylated-ghrelin has various physiological functions via unknown specific receptor, including involvement in cell death, feeding behavior, and energy and glucose homeostasis [8–13]. Moreover, in 2005, obestatin was identified as a peptide hormone derived from preproghrelin in the stomach [14] and obestatin has been shown to be involved in energy homeostasis, gastrointestinal motility, memory, sleep, and cell proliferation [13–18]. 

In this review, we focus on ghrelin cells in the gastrointestinal tract and describe the characteristic features, development, and regulation of ghrelin cells. 

## 2. Distribution and Morphological Features of Ghrelin Cells in the Gastrointestinal Tract

**Figure 1:**
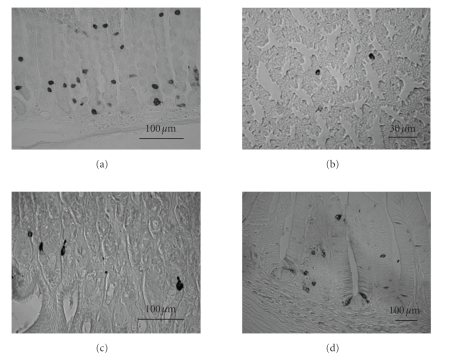
Localization of ghrelin-immunopositive cells in the stomach (rodents, avians, amphibians and fish). (a) Ghrelin cells in the rat stomach. Ghrelin cells are mainly observed from the glandular base to the body of the fundic gland, and gastric ghrelin cells in the rat have been clarified to be closed-type cells. Ghrelin cells are scattered throughout the mucosal layer in (b) the proventriculus of the chicken and (c) bullfrog stomach, and those ghrelin cells are also closed-type cells as rodents. (d) Ghrelin cells in the trout stomach are localized in the mucosal layer, and both closed- and opened-type cells are found in the trout. Scale bars = 100 *μ*m in (a), (c), (d); 30 *μ*m in (b).

The distribution of ghrelin-producing cells in the gastrointestinal tract has been studied by using immunohistochemical techniques and in situ hybridization [1, 2, 19, 20]. In rodents, ghrelin-producing cells were observed in all regions of the gastrointestinal tract: gastric body, antrum, duodenum, ileum, cecum, and colon. Ghrelin-producing cells were most dense in the gastric body (Figure 1(a)) and were found in the mucosal layer but not in the myenteric plexus in all of the examined regions. In the stomach, most of the ghrelin cells were observed in the glandular base decreasing in amount as the gland extends toward the lumen with a few cells observed in the glandular neck. In the duodenum, ileum, cecum, and colon, ghrelin cells were scattered in the epithelia of crypts and villi. The densities of ghrelin cells were dramatically decreased toward the lower gastrointestinal tract [19]. The number of ghrelin-producing cells in each gastrointestinal tract corresponded well with the amount of peptide and mRNA expression levels [21]. In addition, plasma concentration of ghrelin in rats was decreased by 80% after gastrectomy [1], and also plasma ghrelin levels were reduced by 65% in human gastrectomised patients, strongly suggesting that major source of circulating plasma ghrelin is the gastric mucosa [22].

The morphological features of ghrelin-producing cells in the stomach differ from those of the intestinal tracts. In the stomach, ghrelin-producing cells were found to be small and round-shaped, so-called closed-type cells [1, 19, 23]. However, in the duodenum, ileum, cecum, and colon, two types of ghrelin cells were found; that is, closed-type cells with triangular or elongated shapes and opened-type cells with their apical cytoplasmic process contacting to the lumen. In general, opened-type cells are functionally regulated by receiving luminal information such as nutrients and pH, while closed-type cells are functionally modulated by hormones, neuronal stimulation, or mechanical distension [24]. Thus, the regulatory mechanisms of ghrelin release would be different in the stomach and other parts of the gastrointestinal tract. By electron microscopic observation, several groups have shown that immunogold labeling for ghrelin was localized on the round and electron-dense granules in gastric mucosal cells [2, 25–27]. Yabuki et al. showed that the diameters of granules containing ghrelin in mice (277.7 ± 11.1 nm) and rats (268.8 ± 13.0 nm) were similar; however, those in hamsters (200.8 ± 8.8 nm) were significantly smaller than those in mice or rats [26]. Rindi et al. demonstrated that mouse and canine ghrelin-immunoreactive cells closely resembled those of the human stomach, though it has been shown that dog ghrelin cells have obviously larger granules (mean diameter of 273 ± 49 nm) than those of rats (mean diameter of 183 ± 37 nm) and humans (147 ± 30 nm) [27]. 

Localization of GOAT (Ghrelin *O*-Acyltransferase) and ghrelin was also recently studied by using in situ hybridization histochemistry and immunohistochemistry, and a high degree of colocalization of GOAT and ghrelin was observed in mouse gastric oxyntic mucosal cells [28]. On the other hand, ghrelin-GFP transgenic mice expressing humanized *Renilla reniformis* green fluorescent protein (hrGFP) under control of the mouse ghrelin promoter has recently been generated and it has been confirmed that hrGFP expression was especially abundant in the gastric oxyntic mucosa, in a pattern mirroring that of ghrelin immunoreactivity and ghrelin mRNA [28]. 

## 3. Relationship of Ghrelin and Motilin Cells

Motilin was originally isolated from porcine intestinal mucosa in the 1970s [29, 30]. Motilin is 22-amino-acid peptide and it is known to play an important role for the interdigestive migrating motor complex (MMC) [31]. Ghrelin and motilin and their receptors have similar structures and these two peptides are known to form a motilin-ghrelin peptide family [5, 32]. It has been demonstrated that ghrelin stimulates gastrointestinal motility in rodents, suggesting that a part of the physiological effects of these two peptides is also overlapped. The distributions of ghrelin and motilin cells are separated; motilin is mainly produced in the upper intestine, but ghrelin cells are localized mainly in the stomach. Recently, Wierup et al. demonstrated that over 90% of ghrelin cells in the porcine duodenum coexpressed motilin, and they also observed by electron microscopic immunolabeling that they were localized on the same secretory granules, indicating that they were cosecreted with the same stimulation [33]. On the other hand, sequences of ghrelin and motilin in the house musk shrew were determined, and no coexistence of ghrelin and motilin was found in gastric and duodenal cells [34, 35]. Therefore, further studies are needed to elucidate the coexistence of ghrelin and motilin in one cell by using other animal models. 

## 4. Development of Ghrelin Cells in the Gastrointestinal Tract

Ghrelin was found to be expressed in the fetal stomach from embryonic day 18 and the number of fetal gastric ghrelin cells increased as the stomach grew, with gastric ghrelin content also increasing with advance of age [36]. Detailed study showed that ghrelin-immunopositive cells appeared in the glandular base of the fundic gland at 1 week of age and they were found in the glandular base and the glandular neck at 3 weeks of age. Then the distribution of ghrelin cells was extended from the glandular base to the glandular neck during the postneonatal developmental period [37]. Walia et al. have also reported that ghrelin-immunoreactive cells were rare at embryonic day 21 and that their number increased progressively until weaning [38]. Gastric ghrelin mRNA levels also increased in an age-dependent manner similar to the number of ghrelin cells [36, 37, 39]. In addition, ghrelin cells in female rats differentiated at an earlier stage of development than that in male rats, and the density of ghrelin cells in female rats was also higher than that in male rats [37]. In humans, plasma ghrelin level was also higher in females than in males [40]. 

## 5. Distribution and Features of Ghrelin Cells in Nonmammalian Vertebrates

Ghrelin has been characterized not only in mammals but also, many other species, avians, amphibians, reptiles, and fish, and it has been shown that the structures of ghrelin peptide were well preserved from mammals to lower vertebrates [41]. The N-terminal regions of the first seven amino acids in lower vertebrates were well conserved, and the third serine or threonine residue is modified with medium chain fatty acid, mainly octanoic acid, as in mammals [41]. In nonmammalians, it has been confirmed that ghrelin mRNA is abundantly expressed in the stomach [42–48]. In avians, ghrelin-immunopositive cells were found in the mucosal layer of the proventriculus (Figure 1(b)) that corresponds to a first glandular part of the stomach in which digestive enzymes are mixed with food before the gizzard [49]. However, ghrelin immunoreactivity in avians was not located in the myenteric plexus, and many more ghrelin-immunopositive cells were found in the middle layer than in the base of the mucosal layer, with the majority of ghrelin cells being round-shaped and closed-type cells [49]. Features of gastric ghrelin cells in amphibians and reptiles are similar to mammals or avian ghrelin cells. Ghrelin cells in these animals were also found in the gastric mucosal layer but not in the myenteric plexus or muscle layers of the stomach, and the ghrelin cells are closed-type cells in frogs (Figure 1(c)) and turtles [45, 50]. Ghrelin cells in rainbow trout were found to be localized as closed-type cells and opened-type cells in the gastric mucosa (Figure 1(d)) [51].

## 6. Regulation of Ghrelin Cells in the Gastrointestinal Tract

### 6.1. Ghrelin Cells on Fasting

Ghrelin is a potent orexigenic peptide that stimulates food intake and body weight gain, suggesting that ghrelin plays an important role in energy homeostasis. Numerous data have shown that plasma ghrelin levels were elevated in a fasting state and returned to basal levels after refeeding [32, 52, 53]. On the other hand, peptide content of ghrelin in the stomach decreased after fasting [54], indicating that cytoplasmic ghrelin released from gastric ghrelin cells caused an increase in plasma ghrelin levels. Compared to biochemical analysis, studies on changes in the number and staining property of gastric ghrelin cells in a fasting state are quite restricted. For example, the number of ghrelin-immunopositive cells significantly increased in rats during fasting for 7 days, and then the cell number decreased to a normal level after refeeding [55]. In bullfrogs, no significant differences in numbers of ghrelin cells were observed during fasting for 20 days [50]. Moreover, in fish fasted for 72 hours, Govoni et al. found no clear differences in ghrelin cell numbers and their immuno-staining intensity [18, 30, 56]. Therefore, further studies are needed to elucidate the number of ghrelin cells in a multiple time point study during a fasting period. 

### 6.2. Ghrelin and Nutrients

As mentioned above, ghrelin is known to be a hunger signal from peripheral tissues, and plasma ghrelin levels drop to basal levels after a meal, suggesting that ghrelin cells are regulated by nutrient uptake. It has been reported that oral and intravenous glucose administration sharply reduced plasma ghrelin concentration in rodents [53, 57], and this effect of glucose on ghrelin inhibition was similar to that found in human studies [58–61]. Williams et al. studied the effect of intragastric load of glucose on plasma ghrelin levels, and they found that plasma ghrelin level was reduced by approximately 50% under a normal gastric emptying condition [62]. On the other hand, plasma ghrelin levels were not changed in pyloric cuff-implanted rats, indicating that ghrelin release from gastric ghrelin cells is affected by glucose that is absorbed from the intestine [62]. In addition to glucose, duodenal and jejunal infusions of lipids or amino acids reduced ghrelin levels in rats [63]. These effects have been confirmed in human studies; free fatty acids or protein administration decreased plasma ghrelin levels [64, 65]. However, the mechanisms by which these nutrients regulate on gastric ghrelin release in the stomach remain unclear. Further studies are needed to elucidate the molecular pathway of ghrelin cell regulation by nutrients. 

### 6.3. Hormonal Control of Gastric Ghrelin

Ghrelin is also regulated by circulating and gastric hormones. Ghrelin cells are located close to somatostatin-producing D cells [66], and somatostatin inhibits ghrelin secretion in rats and humans [66–69]. Intravenous administration of glucagon caused transient increases in both acyl- and des acyl-ghrelin levels. Moreover, ghrelin secretion from the perfused stomach was stimulated by glucagon treatment in a dose-dependent manner [70], and this effect was shown to be mediated by glucagon receptors on ghrelin cells [71]. de la Cour et al. performed a microdialysis study to find a possible ghrelin regulator in the stomach, and they found that epinephrine, norepinephrine, endothelin, and secretin stimulated ghrelin release [69]. 

Steroid hormone is also involved in ghrelin regulation. A role of estrogen in the regulation of gastric ghrelin has also been suggested by several studies. In humans, estrogen regulates plasma ghrelin concentration [72–75]. The levels of gastric ghrelin mRNA and plasma ghrelin and the number of ghrelin cells were found to be transiently increased by ovariectomy in female rats [76]. Treatment of gastric mucosal cells with estrogen showed that estrogen stimulated ghrelin expression and ghrelin secretion [77, 78]. These findings indicate that gastric estrogen plays an important role in the regulation of gastric ghrelin at the transcriptional level. 

## 7. Ghrelin Cells in the Digestive Diseases

In addition to the physiological, biochemical, and structural studies of ghrelin, pathological aspects of ghrelin have been studied in various diseases, such as anorexia nervosa, Prader Willi Syndrome (PWS), Polycystic Syndrome (PCOS), and Celiac Disease (CD) [79–84]. The role of ghrelin in Helicobacter pylori (H. pylori) infections, gastric diseases such as chronic gastritis, gastric and duodenal ulcers, and gastric carcinomas, has been widely studied. While there are conflicting results, many papers have shown that plasma ghrelin concentrations in the H. pylori positive subjects were lower than in H. pylori negative subjects [85–87]. When correlated with the plasma ghrelin levels, the number of ghrelin immunopositive cells was significantly lower in H. pylori positive subjects than in negative subjects [85, 88]. Furthermore, ghrelin mRNA expression levels were also lower in H. pylori-infected human subjects [88]. Using Mongolian gerbils as an animal model of H. pylori infection, Suzuki et al. showed that ghrelin mRNA expression levels were significantly lower in animals with H. pylori, compared to control animals [89]. Furthermore, the number of ghrelin immunopositive cells decreased in H. pylori-infected groups 17 and 23 weeks after infection, while the total number of gastric mucosal cells remained unchanged. In addition, when compared to control animals, gastric ghrelin contents were significantly decreased in infected animals 17 and 23 weeks after H. pylori inoculation, although no differences were detected after only four weeks. Notably, ghrelin cells in the gastric mucosa are located close to the parietal cells, and some of these cells are found to be in contact with each other [77, 89], and it has been reported that H. pylori affected the function of parietal cells [90, 91]. Although further studies are needed to elucidate the biological mechanisms behind the regulation of gastric ghrelin after H. pylori infection, it is possible that gastric estrogen, which is produced in parietal cells, could affect ghrelin cells in a paracrine manner. Alternatively, inflammatory factors may act directly on ghrelin cells to decrease their activity. 

## 8. Conclusions

Ghrelin is predominantly produced in the stomach, and gastric ghrelin is thought to play important multiple physiological roles in peripheral signaling. Results of the analysis of ghrelin cells in the gastrointestinal tract have been accumulating. Many factors including nutrients as well as circulating and gastric hormones were found to be involved in the regulation of mRNA and release of ghrelin, indicating that ghrelin cells are under the control of complicated mechanisms, which are still obscure. In addition, characteristics of ghrelin cells in the stomach and other parts of the gastrointestinal tract are different; closed-type ghrelin cells are localized in the stomach and many opened-type ghrelin cells are localized throughout the small and large intestines. Since the length of the gastrointestinal tract is long, considerable amounts of plasma ghrelin can be assumed to be derived from ghrelin cells in small and large intestines. Further studies are needed to understand the physiological and pathological roles of ghrelin cells in both small and large intestines. Moreover, posttranscriptional modifications are very important for physiological active ghrelin, necessitating the need to understand how GOAT is involved in the regulation of ghrelin biosynthesis and consequently may be important for controlling ghrelin cells in some physiological states.
